# ASA Status, *NPPA/NPPB* Haplotype and Coronary Artery Disease Have an Impact on BNP/NT-proBNP Plasma Levels

**DOI:** 10.3390/cells11050766

**Published:** 2022-02-22

**Authors:** Markus Hahn, Ulrike M. Stamer, Markus M. Luedi, Malte Book, Heinz U. Rieder, Frank Stüber

**Affiliations:** 1 Department of Anaesthesiology and Pain Medicine, Inselspital, Bern University Hospital, University of Bern, 3010 Bern, Switzerland; markus.hahn@luks.ch (M.H.); ulrike.stamer@dbmr.unibe.ch (U.M.S.); book.malte@klinikum-oldenburg.de (M.B.); heinz.rieder@insel.ch (H.U.R.); frank.stueber@insel.ch (F.S.); 2 Department of Intensive Care Medicine, Lucerne Cantonal Hospital, 6000 Lucerne, Switzerland; 3 Department for BioMedical Research (DBMR), University of Bern, 3010 Bern, Switzerland; 4 Department of Anesthesia, Intensive Care Medicine, Emergency Medicine and Pain, Therapy, University Hospital, Klinikum Oldenburg AöR, 26133 Oldenburg, Germany

**Keywords:** natriuretic peptide, haplotypes, cardiac risk, perioperative

## Abstract

Plasma concentrations of natriuretic peptides (NP) contribute to risk stratification and management of patients undergoing non-cardiac surgery. However, genetically determined variability in the levels of these biomarkers has been described previously. In the perioperative setting, genetic contribution to NP plasma level variability has not yet been determined. A cohort of 427 patients presenting for non-cardiac surgery was genotyped for single-nucleotide polymorphisms (SNPs) from the NPPA/NPPB locus. Haplotype population frequencies were estimated and adjusted haplotype trait associations for brain natriuretic peptide (BNP) and amino-terminal pro natriuretic peptide (NT-proBNP) were calculated. Five SNPs were included in the analysis. Compared to the reference haplotype TATAT (rs198358, rs5068, rs632793, rs198389, rs6676300), haplotype CACGC, with an estimated frequency of 4%, showed elevated BNP and NT-proBNP plasma concentrations by 44% and 94%, respectively. Haplotype CGCGC, with an estimated frequency of 9%, lowered NT-proBNP concentrations by 28%. ASA classification status III and IV, as well as coronary artery disease, were the strongest predictors of increased NP plasma levels. Inclusion of genetic information might improve perioperative risk stratification of patients based on adjusted thresholds of NP plasma levels.

## 1. Introduction

Standardization of care and cardiovascular risk stratification have been widely implemented to improve the perioperative safety of patients [1]. The natriuretic peptides (NP), especially brain natriuretic peptide (BNP) and N-terminal prohormone of brain natriuretic peptide (NT-proBNP), are established biomarkers for cardiac derangement, with numerous studies demonstrating the prognostic and diagnostic value of NP [2,3] and meta-analyses confirming the validity of NPs as biomarkers for perioperative risk stratification [2,4,5,6,7,8,9,10]. Some international guidelines recommend NP testing in the perioperative setting [1,11], and the Canadian Cardiovascular Society guidelines on perioperative cardiac risk assessment give clear-cut thresholds of NP plasma levels in patients at moderate to high risk of perioperative cardiovascular complications [11]. In a large international multi-center cohort enrolling 10,402 patients, preoperative NT-proBNP was strongly associated with vascular death and myocardial injury within 30 days after non-cardiac surgery [12]. However, significant individual variability in NP concentrations has been described [13]. The natriuretic peptide precursor B (NPPB) gene encodes BNP, and the adjacent natriuretic peptide precursor A (NPPA) gene influences plasma concentrations. Studying a Japanese cohort, Takeishi et al. identified two haplotypes in the *NPPA/NPPB* locus correlating with BNP plasma levels (TTGCAT and CCATGC for SNPs rs6676300, rs198389, rs198388, rs6668352, rs198375, and rs632793) [14]. Furthermore, several single-nucleotide polymorphisms (SNP) in this locus have been shown to be associated with NP plasma levels [15,16,17,18]. However, the relevance of these genetic variants and their influence on NP plasma concentration for preoperative risk stratification is unknown. *NPPA/NPPB* haplotype composition and potential correlation with NP plasma levels has not been studied in a preoperative surgical cohort that might differ from a random sample of the general population by underlying factors associated with the genetic background on the one hand, and the need for surgery on the other. In order to investigate the impact of genetic variation on the predictive value of NPs, we hypothesized that common haplotypes in the *NPPA/NPPB* locus influence NP plasma levels in a cohort of patients scheduled for non-cardiac surgery.

## 2. Materials and Methods

### 2.1. Study Design and Patients

We conducted a genetic association study to investigate the association between *NPPA/NPPB* haplotypes and NP plasma concentrations. The Cantonal Ethics Committee (Kantonale Ethikkommission Bern, Switzerland, Chairperson Prof C. Seiler; KEK 041/09) provided ethics approval for this prospective single-center study. The research protocol is registered at ClinicalTrials.gov (identifier NCT04327258). Patients undergoing elective non-cardiac surgery at the Bern University Hospital in Switzerland over a 22 months‘period were screened for eligibility and enrolled in the study after giving written informed consent. The study was designed as a two-group comparison enrolling a cohort of younger and healthy patients (age 39–50 years, ASA (American Society of Anesthesiology) physical status classification [19] I and II), and an older cohort eventually carrying cardiac risk factors (age 50 years and older, ASA III or IV). To assure the power of the analysis allowing for reliable haplotype determination, the analysis combined the cohorts for a fully likelihood-based approach, by which simultaneous estimation of haplotype frequency and haplotype-trait associations was possible [20,21].

Exclusion criteria included cardiac surgery, language barriers, cognitive impairment, substance use disorder, and psychiatric disease. Reasons for exclusion from the analysis were missing NT-proBNP measurements and genotype information for any SNPs under investigation. Participants with missing data other than NP concentrations and genotype were not excluded listwise but for analyses involving the respective variable. 

Patient characteristics (sex, age, weight), medical history (coronary artery disease, cerebrovascular disease, insulin-dependent diabetes mellitus), serum creatinine as well as NYHA (New York Heart Association) functional classification and ASA classification were obtained during the patients’ preoperative anesthetic evaluation and were recorded using a standardized form. 

### 2.2. BNP and NT-proBNP Assay

Blood was collected during induction of anesthesia (2 EDTA tubes with a filling volume of 2.7 mL each, one lithium-heparin tube with a filling volume of 4.7 mL) and transferred immediately to the department of laboratory medicine. The samples were processed and analyzed using routine diagnostics for plasma BNP (Abbott Laboratories, Green Oaks, IL, USA) and NT-proBNP concentrations (Roche, Rotkreuz, Switzerland). Blood cells were frozen at minus 20 °C for genetic analysis.

### 2.3. Genetic Analysis

SNPs either involved in the regulation of BNP/NT-proBNP or localized inside the *NPPA/NPPB* gene locus were chosen as candidates [14,15,16,17,18,22]. The following SNPs were selected: rs198389, rs198358, rs5063, rs5068, rs6676300, rs11079028, rs632793, and rs12562952.

DNA was extracted from whole blood using the Reliaprep™ blood gDNA miniprep kit (Promega, Madison, WI, USA) according to the manufacturer’s instructions. In brief, cells were homogenized and lysed, and DNA was separated using a binding column. Purified DNA was eluted in 200 µL water, and the concentration was measured on a Nanodrop™ 2000 (ThermoFisher Scientific, Waltham, MA, USA).

Eight single-nucleotide polymorphisms were analyzed using hybridization probes labeled with FAM and ATTO620 dyes (Microsynth, Balgach, Switzerland) and either LightCycler^®^ FastStart DNAMaster HybProbe or LightCycler^®^ 480 Genotyping Master Mastermix (Roche, Rotkreuz, Switzerland) on a LightCycler^®^ 480 II system (Roche, Rotkreuz, Switzerland). Primer sequences for SNP genotyping with corresponding annealing temperatures are displayed in Supplementary Table S1. Primers were used at a concentration of 20 µM (Promega, Madison, WI, USA) and probes at a concentration of 2 µM. Each real-time PCR was performed as follows: 95 °C for 10 min; 45 cycles of 95 °C for 10 s, primer-specific annealing temperature for 10 s, 72 °C for 10 s. Subsequently, a melting program was run from 40 °C to 80 °C and a cooling step to 30 °C. Analysis was performed using the software LightCycler^®^ 480 version 1.5.0 and the built-in T_m_ calling program for second derivative peak analysis. 

Adherence of genotype and allele frequencies to the Hardy-Weinberg Equilibrium (HWE) was tested with the genetics library [23]. SNPs that did not meet the HWE were excluded from subsequent analyses, as violation of HWE can be due to genotyping errors.

### 2.4. Statistical Analysis

Haplotype frequencies at the population level were estimated using an expectation–maximization algorithm from the haplo.stats library [24], with inclusion of all allele loci and a trimming threshold of zero. For descriptive analysis, medians and interquartile ranges (IQR) of NP plasma concentrations across levels of categorical covariates were calculated. BNP and NT-proBNP concentrations for single SNP genotypes were compared with the Kruskal-Wallis test. A Bonferroni correction of the alpha level for significance was applied for single SNP genotype effects to account for multiple testing. In case of significant differences across genotypes, a pairwise post-hoc analysis was conducted. 

To assess the proportion of carriers of a specific haplotype displaying elevated NP concentrations associated with an increased risk of death or myocardial infarction, cut-off values as published in the Guidelines of the Canadian Cardiovascular Society (BNP ≥ 92 ng l^−1^; NT-proNP ≥ 300 ng l^−1^) were used [11]. To estimate the influence of common haplotypes on risk estimation, these cut-offs were adjusted to the modifying effect of the respective haplotype. 

Haplotype-trait associations were estimated with the haplo.glm function [24], involving simultaneous estimation of haplotype frequency and haplotype-trait associations within a generalized linear model. As the endpoint of the analysis, NP plasma concentrations were set up as dependent variables for the regression models. BNP and NT-proBNP measures were log-transformed to account for right-skewed distributions. An additive genetic model was assumed. For haplotype frequencies, a cut-off >0.02 was applied for inclusion in the model. Haplotypes with lower frequencies were combined in the rare haplotype category. Covariates included were sex, age, weight, history of coronary artery disease, history of cerebrovascular disease, history of insulin-dependent diabetes mellitus, plasma creatinine levels as well as NYHA class and ASA classification. Patients with known heart failure were classified according to the severity of their symptoms into NYHA classes I to IV. Patients without heart failure were assigned a value of zero. The choice of covariates was motivated by either their influence on NP plasma concentrations or their association with perioperative cardiovascular complications.

Statistical analysis was carried out with R (R Core Team, R Foundation for Statistical Computing, Vienna, Austria. URL https://www.R-project.org/, accessed on 1 October 2021). Statistical significance was set at a *p*-value < 0.05.

## 3. Results

**Figure 1:**
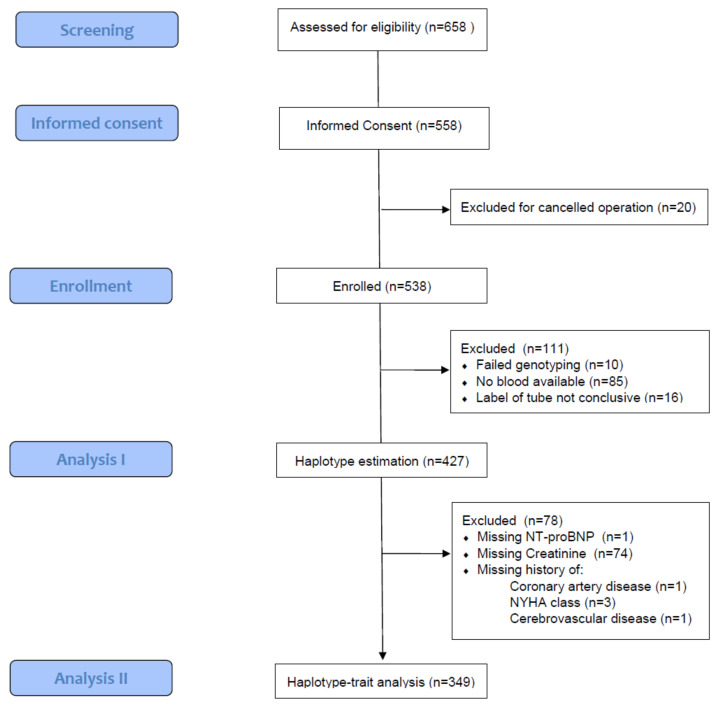
Flowchart of inclusion and exclusion processes.

Six hundred and fifty-eight patients were screened and 427 were analyzed (Figure 1). 

The number of missing observations for covariates was 77 and one for NT-proBNP. These cases were excluded from haplotype-trait analysis, thus resulting in 349 patients for the analysis regarding NT-proBNP and 350 patients for the analysis of BNP. Allele frequencies of the SNPs investigated are displayed in Supplementary Table S2. The Hardy–Weinberg equilibrium was not met for rs5063, rs11079028 and rs12562952, which were excluded from the analysis. 

### 3.1. Descriptive Statistics

#### 3.1.1. BNP and NT-proBNP Distributions

Values for BNP and NT-proBNP generally skewed to the right, with medians of 24 ng l^−1^ (IQR 12–56) for BNP and 67 ng l^−1^ (IQR 33–167) for NT-proBNP. Sixty-two patients exhibited elevated BNP values (≥92 ng l^−1^), with three of them being preoperatively categorized as ASA class I or II (1.5% of this group), and 59 (26.9%) were ASA III or IV. For NT-proBNP, 65 patients exhibited elevated NT-proBNP values (≥300 ng l^−1^). They were all ASA III or IV (29.7%).

#### 3.1.2. BNP and NT-proBNP Concentrations by Level of Covariates

Plasma concentrations of BNP and NT-proBNP across levels of categorical covariates are shown in Table 1. 

Higher BNP and NT-proBNP concentrations were seen in patients with higher ASA classification, higher NYHA class, in females compared to males, and in patients with comorbidities (coronary artery disease, cerebrovascular disease, and insulin-dependent diabetes mellitus). Supplementary Table S3 provides *p*-values for univariate testing between levels of categorical variables.

### 3.2. Genetic Effects on Natriuretic Peptides

#### 3.2.1. Single-SNP Effects on NP Plasma Levels

Effects of single SNP genotypes on BNP and NT-proBNP levels are displayed in Table 2.

**Figure 2:**
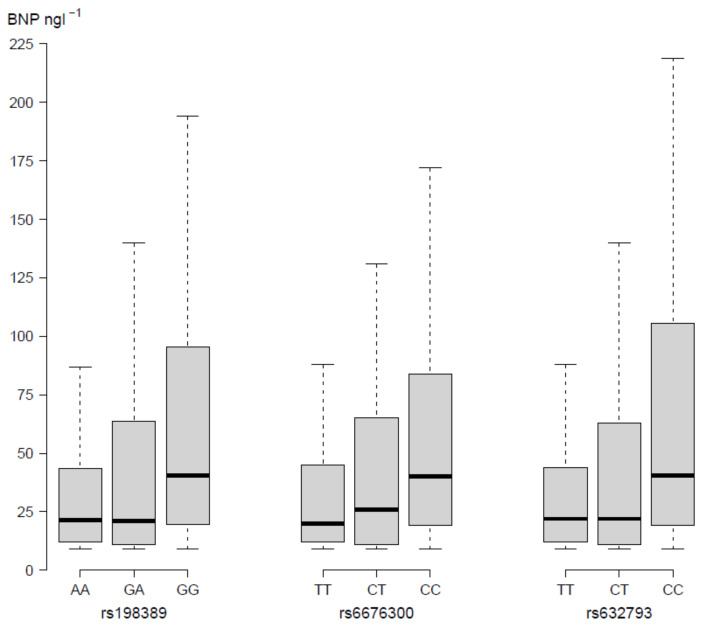
Effect of single SNP genotypes on BNP plasma concentrations for rs198389, rs6676300 and rs632793. Abbreviations: A = adenine; G = guanine; T = thymine; C = cytosine; BNP = brain natriuretic peptide. A Kruskal–Wallis test was used for assessment of significance, with Bonferroni correction (10 tests, level of significance 0.005). Box and whiskers represent medians, IQR and Q1 − 1.5 × IQR and Q3 + 1.5 × IQR.

Three SNPs were shown to influence BNP, but not NT-proBNP plasma concentrations. Variant homozygotes for rs198389, rs6676300 and rs632793 had higher median BNP plasma concentrations than heterozygotes and wild types (Figure 2). *p*-values for post-hoc tests are available in Table S4.

#### 3.2.2. Haplotype Organization in the NPPA/NPPB Locus

Haplotype estimation yielded ten haplotypes with inferred population level frequencies of at least 0.01. Three haplotypes covered 80% of haplotype variability (Table 3). Haplotype 9 (TATAT rs198358, rs5068, rs632793, rs198389, rs6676300) was estimated to be present in 53% of the population, haplotype 7 (TACGC) in 18%, and haplotype 2 (CGCGC) in 9%.

#### 3.2.3. Common Haplotypes in the NPPA/NPPB Locus Influence NP Plasma Levels

**Figure 3:**
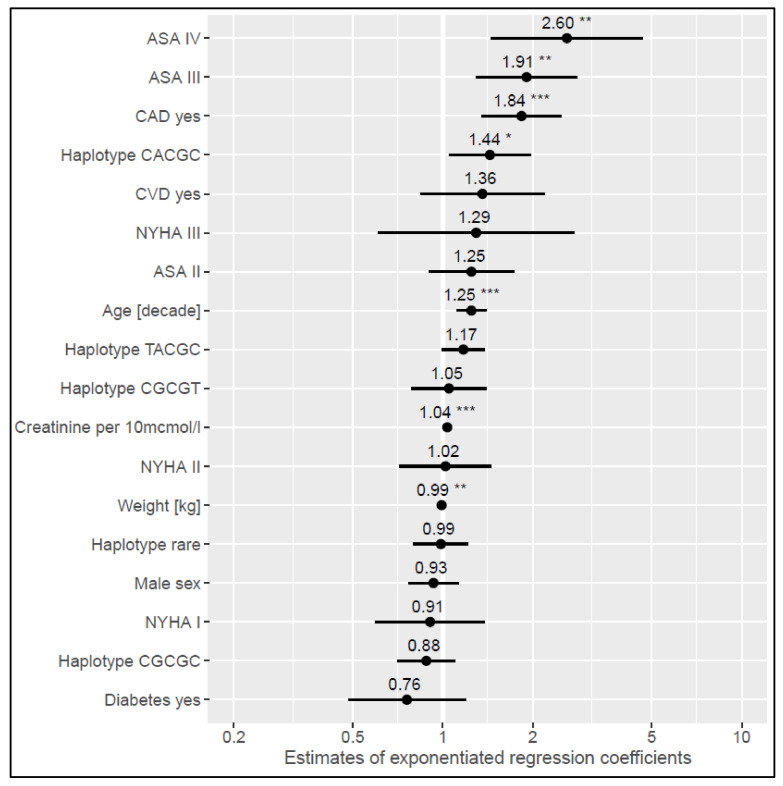
Estimates of exponentiated generalized linear model coefficients to model haplotype effects on logarithmized BNP. Abbreviations: ASA = American Society of Anesthesiologists; CAD = coronary artery disease; NYHA = New York Heart Association; CVD = cerebrovascular disease. * *p* < 0.05, ** *p* < 0.01, *** *p* < 0.001. Reference levels of factors are: Haplotype TATAT, CAD no, NYHA 0 (no heart failure), CVD no, Diabetes no, female sex.

**Figure 4:**
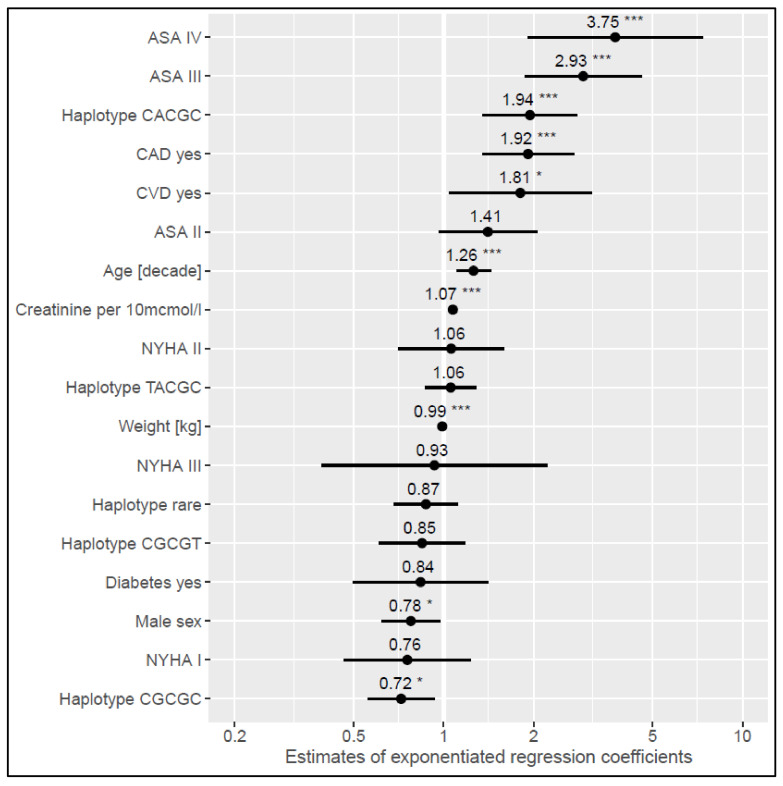
Estimates of exponentiated generalized linear model coefficients to model haplotype effects on logarithmized NT-proBNP. Abbreviations: ASA = American Society of Anesthesiologists; CAD = coronary artery disease; NYHA = New York Heart Association; CVD = cerebrovascular disease. * *p* < 0.05, *** *p* < 0.001. Reference levels of factors are: Haplotype TATAT, CAD no, NYHA 0 (no heart failure), CVD no, Diabetes no, female sex.

Two generalized linear models were developed to model haplotype effects on logarithmized BNP and NT-proBNP. Haplotype 1 (CACGC for rs198358, rs5068, rs632793, rs198389, rs6676300) was shown to elevate BNP plasma levels by 44% (*p* = 0.022) (Supplementary Table S5) and plasma levels of NT-proBNP by 94% (*p* = 0.000) (Supplementary Table S6). Haplotype 2 (CGCGC for rs198358, rs5068, rs632793, rs198389, rs6676300) had a lowering effect of 28% on NT-proBNP plasma levels (*p* = 0.013) (Supplementary Table S6). Exponentiated regression coefficients with corresponding 95% confidence intervals are shown in Figure 3 and Figure 4. Next to the haplotype effects, which were the subject of this analysis, both models showed ASA class III and IV corresponding to the largest effect on NP plasma levels. The influence of other variables and their magnitude in relation to the haplotype effects can be obtained from Figure 3 and Figure 4.

### 3.3. Influence of Common Haplotypes on Risk Estimation

Thirty-four patients were carriers of at least one CACGC haplotype as part of the haplotype pair with the highest estimated posterior probability. Of these, six patients showed elevated BNP levels exceeding the 92 ng l^−1^ recommended by the Canadian guidelines on preoperative risk evaluation, and 10 patients showed NT-proBNP concentrations exceeding the 300 ng l^−1^. Accounting for the effect of the CACGC haplotype, six patients might have been mistakenly classified to be at increased perioperative cardiovascular risk if only considering their elevated NP values (one patient with BNP and five with NT-proBNP concentrations above the cut-off values) and not counting the effect of the underlying genotype. 

Seventy-four patients had at least one CGCGC haplotype as part of the haplotype pair with the highest estimated posterior probability. Of these, 63 patients showed NT-proBNP levels below the threshold of 300 ng l^−1^. Accounting for the effect of the CGCGC haplotype, one patient might have been incorrectly classified as low risk for perioperative cardiovsascular events due to the effect of his underlying genotype resulting in decreased NT-proBNP concentrations. 

The cut-off values corrected for the haplotype effect were 133 ng l^−1^ for BNP and 585 ng l^−1^ for NT-proBNP for the CACGC haplotype and 216 ng l^−1^ for NT-proBNP for the CGCGC haplotype.

## 4. Discussion

In this cohort comprising 349 preoperative patients, we showed that two common haplotypes in the *NPPA/NPPB* locus, with population-level frequencies estimated as 4% and 9%, respectively, were associated with BNP (Haplotype 1) and NT-proBNP (Haplotypes 1 and 2) concentrations. Furthermore, NP plasma concentrations were associated with known confounders in generalized linear models. ASA class III and IV, as well as coronary artery disease, were shown to have the largest effect. To our knowledge, this is the first study to investigate genetic variability in NP plasma levels and NPPA/NPPB haplotypes in a Swiss cohort presenting for non-cardiac surgery. 

In a Japanese cohort, Takeishi and coworkers found two risk haplotypes in the *NPPA/NPPB* locus to be associated with BNP plasma levels [14]. Although the haplotypes differ from those in the present trial with regard to included SNPs, there is overlap by three SNPs: rs632793, rs198389 and rs6676300. Haplotype 1 from the present cohort aligns with haplotype 2 from the Japanese cohort. The haplotype effect is positive for BNP plasma concentrations in both studies, whereas the association with NT-proBNP plasma concentrations was only investigated in the present trial. 

It is still unclear whether elevated NP levels are indicators of physiological derangement, or if individuals who are genetically prone to a stronger release of NP actually compensate more efficiently for cardiac stress. Several studies have reported on SNPs that were associated both with elevated NP plasma levels and with decreased blood pressure, indicating a protective effect [25,26,27,28]. Nevertheless, there is conflicting evidence of diagnostically meaningful association of genotype with outcome. In some studies, associations between genetic variants in the *NPPA/NPPB* locus and various cardiovascular outcomes (rs5063 with blood pressure progression [29], rs198389 with risk of diabetes mellitus type 2 [18], cardiovascular mortality and lifespan [28]) have been reported. In contrast, other working groups did not find associations of rs198389 with a composite outcome of myocardial infarction, stroke, or cardiovascular death [30], of rs5063, rs198358 and rs632793 with the prognosis of kidney damage [16], and of rs198389, rs5063 and 198358 with a relevant risk of heart failure [31]. 

Fox and co-authors reported on a decreased incidence in postoperative ventricular failure after coronary artery bypass grafting in patients carrying minor alleles of rs6676300 and rs198389 [32]. In general, previous investigations on the predictive value of pre- and postoperative NP plasma concentrations have not included genetic information so far [2,4,6,7,8,9,10,33].

In the preoperative setting, the negative predictive value of non-elevated plasma concentrations of NPs is pointed out [7]. Therefore, the association of haplotype 2 with lower plasma concentrations of NT-proBNP—if proven to be replicable—could result in misclassifying patients as at low risk due to their haplotype. This is especially relevant for asymptomatic patients with no known underlying cardiac condition. In these patients, NP concentrations are an important tool to detect possible, previously unknown cardiovascular co-morbidity during the patients’ preoperative anesthesia evaluation. 

The importance of NP for perioperative risk stratification is low in guidelines from Europe [1] and the United States [34] compared to those from Canada [11]. If the present results can be replicated, the next step would be to investigate the predictive power of genetic information in preoperative risk models. Rethinking the thresholds for NP plasma concentrations might be worth considering, as genetic testing becomes increasingly cheaper and even small improvements in the overall performance of risk evaluation could be cost-effective. 

## 5. Limitations

We analyzed a mixed Swiss surgical cohort. However, results are limited by the inherent shortcomings of a single-cohort study. NP plasma levels were measured only once during induction of anesthesia, which might not reflect underlying physiological variability. However, this would result in non-differential misclassification, with a more conservative estimate of the genetic contribution to variability in NP plasma concentrations (bias towards the null). A future strategy of serial measurements could easily be implemented in clinical practice. Patients’ cardiovascular medication was not included in the analysis, which could have introduced confounding. 

Regarding the assays used for NP plasma level measurements: there is a large diversity of available assays with a lack of harmonization across specific products [35]. Therefore, replication with assays other than those used in the present trial could also result in non-differential misclassification. It is worth mentioning that neither a universal approach to standardized assays for NP measurements nor a validation of cut-off values has been established up to now [33]. Additionally, the study was not powered to uncover small effect sizes. For example, we observed a reverse but insignificant single SNP effect of rs5068 compared to other studies [17,25]. Finally, the mixed surgical cohort investigated corresponds to a low cardiovascular risk setting, whereas the implementation of NP-based risk stratification is most powerful in a high cardiovascular risk setting (major abdominal, thoracic or vascular surgery) [1]. Nevertheless, it seems unlikely that the association of genotype and NP plasma levels, if depicting real underlying physiology, would alter based on the type of surgery.

## 6. Conclusions

Two haplotypes in the *NPPA/NPPB* locus appear to be associated with altered levels of NP. If the modulating effects of *NPPA/NPPB* haplotypes on NP plasma concentrations can be confirmed, future approaches to preoperative stratification of cardiac risk can potentially be improved. 

## Figures and Tables

**Table 1:** Distribution of BNP and NT-proBNP across subgroups of categorical covariates.

Covariate	Subgroup	n (%)	BNP [ng l^−1^]	NT-proBNP [ng l^−1^]
Sex	Female	187 (44)	26.0 (14.0, 55.0)	72.0 (43.5, 155.5)
	Male	239 (56)	22.0 (10.0, 56.0)	58.0 (25.0, 195.0)
Coronary artery disease	No	383 (90)	21.0 (12.0, 48.0)	58.0 (30.0, 129.5)
	Yes	42 (10)	**101.5** (40.0, 202.5)	**331.0** (107.2, 597.2)
Cerebrovascular disease	No	410 (96)	22.5 (12.0, 52.5)	65.0 (31.25, 147.5)
	Yes	15 (4)	**97.0** (59.5, 154.0)	**478.0** (218.5, 958.5)
Diabetes mellitus	No	407 (96)	24.0 (12.0, 54.0)	65.0 (32.0, 151.0)
	Yes	17 (4)	48.0 (15.0, 84.0)	236.0 (67.0, 466.0)
ASA physical classification	1	60 (14)	15.5 (9.0, 22.0)	33.5 (21.8, 53.3)
	2	147 (35)	15.0 (9.0, 26.0)	41.0 (21.0, 69.0)
	3	205 (48)	44.0 (21.0, 90.0)	132.0 (66.0, 335.0)
	4	14 (3)	**98.0** (78.5, 163.0)	**446.0** (189.2, 874.2)
NYHA class	0	375 (89)	22.0 (12.0, 51.0)	60.0 (31.0, 141.5)
	1	18 (4)	26.0 (15.0, 76.0)	81.5 (36.0, 176.8)
	2	25 (6)	37.0 (25.0, 102.0)	132.0 (82.0, 289.0)
	3	5 (1)	**126.0** (38.0, 174.0)	248.0 (161.0, 466.0)

Plasma concentrations of BNP (brain natriuretic peptide) and NT-proBNP (N-terminal pro natriuretic peptide) are displayed as median (IQR); ASA = American Society of Anesthesiologists; NYHA = New York Heart Association; **bold** type for median values above the cut-off values as used by the Canadian Cardiovascular Society (BNP ≥ 92 ng l^−1^, NT-proBNP ≥ 300 ng l^−1^). No patients classified as NYHA class 4.

**Table 2:** Effects of single SNP genotypes on plasma levels of BNP and NT-proBNP.

	Genotype (n)	BNP [ng l^−1^]	*p*	NT-proBNP [ng l^−1^]	*p*
rs198358	TT (256)	24.5 (12.0, 56.0)		69.5 (34.0, 196.0)	
	CT (142)	24.0 (11.0, 59.5)		67.5 (28.3, 158.3)	
	CC (28)	21.5 (16.8, 40.8)	0.879	66.0 (44.0, 123.8)	0.562
rs5068	AA (283)	25.0 (13.0, 62.0)		73.0 (35.5, 206.5)	
	AG (126)	22.0 (11.0, 51.0)		58.0 (27.3, 148.0)	
	GG (17)	21.0 (16.0, 32.0)	0.565	66.0 (44.0, 77.0)	0.110
rs632793	TT (152)	21.5 (11.8, 44.3)		62.0 (33.0, 123.8)	
	TC (210)	22.0 (11.3, 62.5)		65.5 (29.0, 166.0)	
	CC (64)	40.5 (19.0, 103.3)	<0.001 *	99.5 (54.8, 346.0)	0.010
rs198389	AA (143)	21.0 (12.0, 43.0)		60.0 (33.5, 125.5)	
	GA (215)	21.0 (11.0, 63.5)		65.0 (27.5, 174.0)	
	GG (68)	40.5 (19.8, 92.6)	<0.001 *	83.5 (56.5, 273.5)	0.015
rs6676300	TT (176)	20.0 (12.0, 45.3)		57.0 (31.8, 123.8)	
	CT (189)	26.0 (11.0, 65.0)		71.0 (29.0, 176.0)	
	CC (61)	40.0 (19.0, 84.0)	0.003 *	82.0 (54.0, 338.0)	0.022

Plasma concentrations of BNP and NT-proBNP are shown as medians (IQR). rs Reference SNP number; SNP = single nucleotide polymorphism; G = guanine; A = adenine; C = cytosine; T = thymine. *: significance level for *p* values is corrected for multiple testing (Bonferroni correction, 10 tests, alpha = 0.005).

**Table 3:** Haplotype organization and frequencies in the NPPA/NPPB locus.

Haplotype	rs198358	rs5068	rs632793	rs198389	rs6676300	Frequency
1	C	A	C	G	C	0.04
2	C	G	C	G	C	0.09
3	C	G	C	G	T	0.05
4	C	G	T	A	C	0.02
5	C	G	T	A	T	0.01
6	C	G	T	G	C	0.01
7	T	A	C	G	C	0.18
8	T	A	C	G	T	0.02
9	T	A	T	A	T	0.53
10	T	A	T	G	C	0.01

Likelihood ratio statistic for no linkage disequilibrium = 1398.215, df = 15, *p* = 0. rs reference SNP number; G = guanine; A = adenine; C = cytosine; T = thymine.

## Data Availability

The dataset in support of the findings of this study is available from the corresponding author, upon reasonable request.

## Supplementary Materials

The following are available online at https://www.mdpi.com/article/10.3390/cells11050766/s1, Table S1: Primer sequences for SNP—genotyping, Table S2: Allele frequencies for genotyped single nucleotide polymorphisms; Table S3: Distribution of BNP and NT-proBNP across subgroups of categorical covariates; Table S4: *p*-values for post-hoc comparisons of single-SNP effects; Table S5: Generalized linear model coefficients to model haplotype effects on logarithmized BNP; Table S6: Generalized linear model coefficients to model haplotype effects on logarithmized NT-proBNP
