# Supplementary material for: ASA Status, NPPA/NPPB Haplotype and Coronary Artery Disease Have an Impact on BNP/NT-proBNP Plasma Levels
Source: Cells. 2022 Feb 22;11(5):766. doi: 10.3390/cells11050766 (PMC8909668; doi:10.3390/cells11050766)
Supplement: Supplementary file 1 [file cells-11-00766-s001.zip › cells-1440206-supplementary.pdf]

Supplemental Material for

"ASA Status, NPPA/NPPB Haplotype and Coronary Artery Disease have an impact on BNP/NT-proBNP plasma levels"

Table S1 Primer sequences for SNP genotyping

reference SNP	forward primer (5'-3')	reverse primer (5'-3')	donor probe (5'-3')	detection probe (5'-3')	*Annealing temperature C°*
rs198389	GGAGACACAGACAAGTCC	CCTCCCTGCCTTTTCC	GCAAATGTCCGGGTGTCCTA-FAM	AT620-TGGCGCTTTCTTCCTGCCTCCC-PHOS	*53*
rs198358	AAATGCCCCTGGGACT	GCTTCTGTTGTTGCCATC	GGATGGATGCAGGAGCTGAACTGGCT-FAM	AT620-CACTGATGGAACGGCCACTTCT-PHOS	*48*
rs5063	CTCCTTCTCCACCACCA	AGCACAGCATCAGAAAGC	GTACAATGCCGTGTCCAACGC-FAM	AT620-TGATGGATTTCAAGGTAGGGCCAGGAAAGCGG-PHOS	*53*
rs5068	AGGGAGGACAAGCAGG	GCGAGGAAGTCACCATC	GGCTCCTGTCCCCTGGGGTCTC-FAM	AT620-TGCATTTGTGTCATCTTGTTGCCATG-PHOS	*55*
rs6676300	TTCATGTCTCTCAACAGCAG	CGGGAGGTGGAGGTTT	GGAGAAAGGATGAGAGGTCCG-FAM	AT620-AATCTCGCCCTATCACCCAGGCTGGA-PHOS	*52*
rs11079028	GCCCTCTGTTTGATCCTT	CCTCGTCAGCTTTGGTC	TCTAATGCTCACACTCCCAACACA-FAM	AT620-TGGGCAGGGAGGCACCATTCAG-PHOS	*60*
rs632793	GGATGATCGTTGCTGACTT	TAACTAAAACAGCCTCTCACTC	TCACTAAGGGCTGAAGACTGGGGACA-FAM	AT620-TTTCCCTAGAAAACAAAGCCCAAGG-PHOS	*54*
rs12562952	CGGCTGAGTCAGGAGT	CCTAGAAGAGGACTGGCA	CTGGGCTGCAATGGCGAGGACAGA-FAM	AT620-AGGCTGGTCCATCGTGTCTCTC-PHOS	*55*

Abbreviations: rs Reference SNP number; SNP = single nucleotide polymorphism; G = guanine; A = adenine; C = cytosine; T = thymine

Table S2. Allele frequencies for genotyped single nucleotide polymorphisms.

SNP	Major Allele	Minor Allele	MAF	p-value HWE*
rs198389	A	G	0.41	0.3622
rs198358	T	C	0.23	0.2216
rs5063	G	A	0.05	0.0033
rs5068	A	G	0.19	0.6446
rs6676300	T	C	0.37	0.4057
rs11079028	C	T	0.24	0.0003
rs632793	T	C	0.4	0.5444
rs12562952	A	G	0.09	0.0439

Abbreviations: rs Reference SNP number; SNP = single nucleotide polymorphism;
G = guanine; A = adenine; C = cytosine; T = thymine; HWE = Hardy Weinberg Equilibrium
MAF = Minor Allele Frequency
*p-values for chi-square test for HWE

Table S3. Distribution of BNP and NT-proBNP across subgroups of categorical covariates

Covariate	Subgroup	n (%)	BNP [ng l^{-1}]	p-value	NT-proBNP [ng l^{-1}]	p-value
Sex	Female	187 (44)	26.0 (14.0, 55.0)		72.0 (43.5, 155.5)	
	Male	239 (56)	22.0 (10.0, 56.0)	0.2600	58.0 (25.0, 195.0)	0.0639
Coronary artery disease	No	383 (90)	21.0 (12.0, 48.0)		58.0 (30.0, 129.5)	
	Yes	42 (10)	**101.5** (40.0, 202.5)	0.0000	**331.0** (107.2, 597.2)	0.0000
Cerebrovascular disease	No	410 (96)	22.5 (12.0, 52.5)		65.0 (31.25, 147.5)	
	Yes	15 (4)	**97.0** (59.5, 154.0)	0.0000	**478.0** (218.5, 958.5)	0.0000
Diabetes mellitus	No	407 (96)	24.0 (12.0, 54.0)		65.0 (32.0, 151.0)	
	Yes	17 (4)	48.0 (15.0, 84.0)	0.1871	236.0 (67.0, 466.0)	0.0215
ASA physical classification	1	60 (14)	15.5 (9.0, 22.0)		33.5 (21.8, 53.3)	
	2	147 (35)	15.0 (9.0, 26.0)		41.0 (21.0, 69.0)	
	3	205 (48)	44.0 (21.0, 90.0)		132.0 (66.0, 335.0)	
	4	14 (3)	**98.0** (78.5, 163.0)	0.0000	**446.0** (189.2, 874.2)	0.0000
NYHA class	0	375 (89)	22.0 (12.0, 51.0)		60.0 (31.0, 141.5)	
	1	18 (4)	26.0 (15.0, 76.0)		81.5 (36.0, 176.8)	
	2	25 (6)	37.0 (25.0, 102.0)		132.0 (82.0, 289.0)	
	3	5 (1)	**126.0** (38.0, 174.0)	0.0047	248.0 (161.0, 466.0)	0.0012

Plasma concentrations of BNP (brain natriuretic peptide) and NT-proBNP (N-terminal pro natriuretic peptide) are displayed as median (IQR); ASA = American Society of Anesthesiologists; NYHA = New York Heart Association; **bold** type for median values above the cut-off values as used by the Canadian Cardiovascular Society (BNP ≥ 92ng l^{-1}, NT-proBNP ≥ 300ng l^{-1}). No patients classified as NYHA class 4. The Kruskal-Wallis Test is used for variables with more than two levels and the Mann-Whitney-U Test for variables with two levels.

Table S4. P-values for post-hoc comparisons of single-SNP effects.

		GA	AA
rs198389	GG	0.0013	0.0002
	GA		1
		CT	TT
rs6676300	CC	0.0372	0.0013
	CT		0.699
		CT	TT
rs632793	CC	0.0029	0.0003
	CT		1

C = cytosine; T = thymine; G = guanine; A = adenine

Table S5. Generalized linear model coefficients to model haplotype effects on logarithmized BNP.

	Coefficient	exp(Coefficient)	Standard Error	T statistic	*P* value
Intercept	2.03	7.61	0.37	5.46	0.000
Haplotype CACGC	0.37	1.44	0.16	2.30	0.022
Haplotype CGCGC	-0.12	0.88	0.11	-1.09	0.278
Haplotype CGCGT	0.05	1.05	0.15	0.36	0.718
Haplotype TACGC	0.16	1.18	0.08	1.94	0.053
Haplotype rare	-0.01	0.99	0.11	-0.07	0.943
ASA class II	0.21	1.24	0.17	1.28	0.202
ASA class III	0.65	1.91	0.20	3.28	0.001
ASA class IV	0.96	2.61	0.30	3.22	0.001
Age (decades)	0.22	1.25	0.06	3.78	0.000
Weight (kg)	-0.01	0.99	0.00	-3.15	0.002
CAD yes	0.61	1.84	0.16	3.91	0.000
NYHA I	-0.09	0.91	0.21	-0.44	0.661
NYHA II	0.02	1.02	0.18	0.13	0.894
NYHA III	0.25	1.29	0.38	0.66	0.507
CVD yes	0.31	1.36	0.24	1.27	0.207
Diabetes yes	-0.27	0.76	0.23	-1.19	0.237
Creatinine per 10 mcmol/l	0.03	1.04	0.01	3.87	0.000
Male sex	-0.08	0.93	0.10	-0.78	0.434

Null deviance: 380.35 (348 df); residual deviance: 210.00 (330 df); AIC: 853.14

Abbreviations: ASA = American Society of Anesthesiologists; CAD = coronary artery disease; NYHA = New York Heart Association; CVD = cerebrovascular disease

Reference levels of factors are: Haplotype TATAT, CAD no, NYHA 0 (no heart failure), CVD no, Diabetes no, female sex

Table S6. Generalized linear model coefficients to model haplotype effects on logarithmized NT-proBNP.

	Coefficient	exp(Coefficient)	Standard Error	T statistic	*P* value
Intercept	2.77	16.02	0.43	6.44	0.000
Haplotype CACGC	0.67	1.94	0.18	3.61	0.000
Haplotype CGCGC	-0.33	0.72	0.13	-2.49	0.013
Haplotype CGCGT	-0.17	0.85	0.17	-0.98	0.327
Haplotype TACGC	0.06	1.06	0.10	0.56	0.576
Haplotype rare	-0.14	0.87	0.13	-1.09	0.275
ASA class II	0.34	1.41	0.19	1.77	0.078
ASA class III	1.08	2.93	0.23	4.72	0.000
ASA class IV	1.32	3.75	0.34	3.84	0.000
Age (decades)	0.23	1.26	0.07	3.41	0.001
Weight (kg)	-0.01	0.99	0.00	-3.46	0.001
CAD yes	0.65	1.92	0.18	3.62	0.000
NYHA I	-0.28	0.76	0.25	-1.13	0.260
NYHA II	0.06	1.06	0.21	0.28	0.779
NYHA III	-0.07	0.93	0.44	-0.16	0.874
CVD yes	0.59	1.81	0.28	2.11	0.036
Diabetes yes	-0.18	0.84	0.27	-0.67	0.505
Creatinine per 10 mcmol/l	0.07	1.07	0.01	6.81	0.000
Male sex	-0.25	0.78	0.11	-2.24	0.026

Null deviance: 612.08 (348 df); residual deviance: 280.57 (330 df); AIC: 954.25

Abbreviations: ASA = American Society of Anesthesiologists; CAD = coronary artery disease; NYHA = New York Heart Association; CVD = cerebrovascular disease

Reference levels of factors are: Haplotype TATAT, CAD no, NYHA 0 (no heart failure), CVD no, Diabetes no, female sex
